# Differences of Symptom Distribution Across Adult Age in High Functioning Individuals on the Autism Spectrum Using Subscales of the Autism Spectrum Quotient

**DOI:** 10.1007/s10803-018-3657-z

**Published:** 2018-07-03

**Authors:** Rob Siebes, Jan-Willem Muntjewerff, Wouter Staal

**Affiliations:** 1 Karakter Child and Adolescent Psychiatry University Centre, Reinier Postlaan 12, 6525 GC Nijmegen, The Netherlands; 2 Department of Psychiatry, Radboud University Medical Centre, Nijmegen, The Netherlands; 3 BuurtzorgT, Nijmegen, The Netherlands; 4 Facculty of Social Sciences, Leiden University, Leiden, The Netherlands

**Keywords:** Autism Spectrum Quotient (AQ), Subscales, Age groups, Adults

## Abstract

Little is known about the distribution of symptoms of Autism Spectrum Disorder (ASD) across the lifespan. In this cross-sectional study, we examined differences between subscales of the Autism Spectrum Quotient (AQ) between different age groups. 654 Subjects referred to an outpatient University Clinic with specialized expertise in ASD were included. Data collection, including self-report and report by spouses, was performed from 2008 to 2014. Results show no significant differences between the different age groups. AQ scores based on self-report corresponded remarkably well with those from their spouses. In conclusion, the main traits of an ASD appear stable between the different age groups. Also, the results show that using the AQ, patients have largely the same appreciation of symptoms as their spouses.

## Introduction

### Studies on Distribution of Symptoms Between Different Age Groups from Child to Adult Life

Autism spectrum disorder (ASD) is a lifelong developmental disability with a prevalence which is hard to measure because of its chronical nature. Earlier prevalence estimates were lower, centering at about 0.5 per 1000 for autism during the 1960s and 1970s as opposed more recent reports of 1–2 per 1000, which may be related to changes in diagnostic practices, referral patterns, availability of services, age at diagnosis, and public awareness (Newschaffer et al. 2007). Symptoms of autism include social and communication impairment and restricted repetitive and stereotyped patterns of behaviour, interests and activities. In a normal population autistic traits are relatively stable from childhood into adulthood (Taylor et al. 2017). The triad of symptoms in people with ASD seems to decrease with age (Magiati et al. 2014), but little is known about the distribution of symptoms between different age groups, both in clinical and non-clinical. Autism in childhood has been well described, but the symptomatology in later age groups has been little subject of investigation, especially for the group of adults with high functioning autism (HFA), or Asperger’s syndrome. It appears that symptom trajectories have considerable individual variation, and should be viewed from a developmental perspective. More at group level, adolescents and young adults may improve more in social interaction than in the Restricted, Repetitive Behaviors and Interests domain (Seltzer et al. 2003).

The ideal model to explore symptom changes over a longer period of time would be a longitudinal study with valid symptom measures. No such study has been performed, which is not surprising given the efforts that it would take. A way to shed some light on this, is to perform a study with cross-sectional symptom measures in different age cohorts. This of course will not provide individual trajectories, but may increase our understanding to some extent. The purpose of this article is to describe the distribution in symptom (clusters) between the different age groups of individuals with HFA using the subscales of the autism quotient (AQ). We hypothesize that the later adult age groups respectively have more problems in repetitive and stereotyped patterns of behaviour than the domains of communication and social skills.

Only few studies investigated the distribution of symptoms of individuals with ASD into adulthood. Reviews on the outcomes of children, adolescent and adults with ASD mainly describe results from studies that are mostly conducted on cognitive and social outcomes (Howlin et al. 2014; Levy and Perry 2011; Magiati et al. 2014; Seltzer et al. 2004). Most of these studies have large variation in IQ scores and small variation in age. They mostly cover a small period in life, and are retrospective using the ADI-R (Autism Diagnostic Interview, Revised). To our knowledge, no studies have been conducted that explore the distribution of symptoms between different adult age groups, which is unfortunate since the clinical importance of providing insight differences in symptomatology between different age groups is evident. The longest follow up study by Howlin et al. (2014), is the only study where a large population of individuals with HFA were followed into late adulthood. This study was mainly focused on cognitive development and social outcomes, but it also describes the development of symptoms after a period of 40 years using the ADI-R. Overall, this study showed a general improvement in autism symptomatology with age. A subgroup which was also assessed 20 years earlier (mean age 26) showed that social outcomes after 40 years of follow up were poorer than the assessment at younger adulthood.

Billstedt et al. (2007) followed a population with a childhood diagnosis of ASD for a period of 13–22 years using the Diagnostic Interview for Social and Communication Disorders (DISCO). A large proportion of the included individuals suffered from intellectual disability. In that study it was found that various types of symptoms in the social interaction category were still common whereas communication problems were much less pronounced on follow-up. Behavioural impairments were much more variable in adulthood. Only one single symptom from this category, maintenance of sameness in routines, was reported to be present in half or more of the study group at the follow-up study.

Apart from these longitudinal studies, several retrospective studies have been published, with mixed designs and patient samples. In a retrospective study by Seltzer et al. (2003) showed that adolescents are more likely to improve in reciprocal social interaction domain than adults, whereas the adults were more likely to improve in the restricted, repetitive behaviours and interests. No differences in severity of symptoms between cohorts in the communication domain were found. However, no levels of IQ were reported in this study. The authors speculated about the possibility that the developmental course of the abnormal behaviours of autism is one of abatement of symptoms from adolescence into adulthood. On the other hand, their adolescent cohort appeared to be less impaired than the adult cohort in the manifestation of prosocial behaviours, such as communication and social interaction.

Other follow up studies have been performed in childhood, including a study by Gillespie-Lynch et al. (2012). It was found that improvements on social domain occur with increased age, but that only minor changes occur with respect to non-verbal communication and repetitive/stereotyped symptoms. Additionally, a study by McGovern and Sigman (2005) suggested improvement in all domains, with high functioning participants showing more extensive improvements. Studies on the transition of adolescence to adulthood found some improvements in restricted repetitive behaviours (Chowdhury et al. 2010). Others (Taylor and Seltzer 2010) found that autism symptoms and maladaptive behaviours were generally improving with age during secondary school, but this improvement slowed down significantly after high school exit.

Overall, it remains unclear whether there are differences of symptom distribution in patients with an ASD, especially in late adulthood. A possibility to investigate this is to use the autism spectrum quotient, which is an efficient instrument for assessing and quantifying autistic traits in individuals.

### Autism Spectrum Quotient (AQ)

The AQ is a questionnaire, which can be used as self-reported or reported by a close relative (AQ-adolescent and AQ-child), was originally developed to identify ASD among adults with normal intelligence (Baron-Cohen et al. 2001). It is translated and validated in Dutch, Japanese, Polish, Australian and Canadian populations (Broadbent et al. 2013; Hoekstra et al. 2008; Lepage et al. 2009; Pisula et al. 2013; Wakabayashi et al. 2006). The AQ contains five theoretically defined subscales of autistic behaviour; social skills, attention switching, attention to detail, communication and imagination.

The AQ consist of 50 items, 10 items per subscale. Original cut-off score (Baron-Cohen et al. 2001) is 32 points, however this cut-off differs per study group. It also quantifies autistic traits in adolescents and children with HFA or Asperger Syndrome (AS) (Auyeung et al. 2008; Baron-Cohen et al. 2006; Sonie et al. 2013). In adolescents and children the AQ is completed by a parent report. For spouses of patients the AQ has not yet been validated yet, although the AQ appears to have high face validity for such a use. One study of Wakabayashi et al. (2006) were the AQ was reduced to a 40 item questionnaire for parents (by 32AS/HFA and parent pairs), shows a mean difference of 2.1 points (SD = 0.5), if the self-reported AQ is compared to the parent reported AQ on these 40 items.

Studies of the use of the AQ in the clinical practice show different results. Woodbury-Smith et al. (2005) found that the AQ is strongly predictive who receives a diagnosis of ASD in adults with AS with normal intelligence and high functioning autism. The cut-off score with the best specificity and sensitivity was 26 out of a total of 50 items. In a Dutch study Sizoo et al. (2015) found that the AQ has no sufficient validity to reliably predict a diagnosis of autism spectrum disorder in outpatient settings.

While the AQ may be less sensitive in HFA for predicting an ASD diagnosis, it appears a valuable tool for assessing and quantifying symptoms of ASD at different ages. In the present study, we therefore used the AQ to measure ASD symptoms at different age stages in order to provide the field with a better understanding of symptom distributions during the lifespan.

Also, the AQ was used to evaluate the appreciation of symptoms of ASD patients compared to their spouses.

## Methods

AQ questionnaires obtained of patients referred to an outpatient University Clinic (Radboud University Hospital, Department of Psychiatry) from 2008 to 2014 were analysed. AQ data, including self-reported and reports by spouses of 878 patients were obtained. 472 spouses filled out the AQ, and 562 patients filled out the self-reported AQ. Of 380 patients both AQ’s where available. Following clinical assessment by a team of ASD experts 654 were diagnosed with an ASD (471 male; 183 female), 195 were between 18 and 30 years of age, 139 between 30 and 40 years, 227 between 40 and 50 years, 151 between 50 and 60 years of age and 65 were older than 60 years of age. DSM-IV classifications were established after a developmental history was taken, in most cases a parent interview, in cases where no parents were available, an interview took place with a close relative. Furthermore an examination by a psychiatrist was performed, and if there still were uncertainties about the diagnosis, an Autism Diagnostic Observation Scheme (ADOS) was performed. The autism quotient questionnaire was a standard instrument used in the diagnostic procedure, both self-reported and parent-reported. Co-morbidity like Attention Deficit Hyperactivity Disorder (ADHD) and personality disorders were not excluded for this study. The distribution between the different subscales and the total score on the AQ of five age groups was investigated (Table 1). The items “attention to detail” and “attention switching” were used to relate to repetitive and stereotyped behavior.

**Table 1:** Mean AQ subscale and total scores by age groups

AQ subscale and total scores by age group Mean (SD)							ANOVA (subscales)	
Age group	18–30	30–40	40–50	50–60	> 60	Total	F	*p*
N	175	125	135	87	40	562		
Males	120	92	104	74	37	427		
Self-reported								
AQ total	29.62 (8.55)	31.98 (7.70)	30.65 (9.02)	30.79 (9.54)	29.90 (10.39)	30.59 (8.80)	1.398	.233
Social skills	6.18 (2.67)	6.81 (2.44)	6.65 (2.61)	6.78 (2.57)	6.23 (3.00)	6.53 (2.62)	1.536	.190
Attention to detail	5.76 (2.53)	5.87 (2.35)	5.53 (2.49)	5.23 (2.35)	5.20 (2.74)	5.61 (2.47)	1.346	.252
Attention switching	7.10 (2.19)	7.60 (2.23)	7.18 (2.40)	6.93 (2.65)	6.83 (2.40)	7.18 (2.34)	1.539	.189
Communication	5.74 (2.43)	6.05 (2.12)	5.74 (2.40)	5.98 (2.72)	5.75 (2.61)	5.85(2.34)	0.478	.752
Imagination	4.84 (2.30)	5.66 (2.21)	5.56 (2.28)	5.87 (2.57)	5.90 (2.38)	5.43 (2.36)	4.403	.002

N	134	109	118	77	34	472		
Males	93	74	85	54	23	329		
Spouses								
AQ total	31.37 (8.30)	33.76 (7.38)	32.42 (7.80)	33.07 (7.00)	34.5 (7.38)	32.69 (7.73)	2.115	.086
Social skills	6.90 (2.50)	7.56 (1.99)	7.06 (2.35)	7.57 (2.12)	7.74 (2.09)	7.06 (7.47)	2.286	.059
Attention to detail	5.21 (2.43)	5.49 (2.61)	5.04 (2.43)	4.75 (2.43)	5.74 (2.48)	4.97 (5.42)	1.508	.199
Attention switching	7.56 (2.13)	8.14 (1.92)	7.77 (2.04)	7.73 (1.94)	7.82 (1.78)	7.61 (7.97)	1.288	.277
Communication	6.08 (2.30)	6.43 (2.23)	6.14 (2.24)	6.55 (2.06)	6.47 (2.19)	6.08 (6.48)	0.853	.492
Imagination	5.61 (2.37)	6.15 (2.33)	6.42 (2.23)	6.45 (2.04)	6.82 (2.49)	5.95 (6.37)	3.333	.010

### Statistical Analyses

All calculations were performed with the SPSS software package, version 23. The means of the different AQ subscales and total AQ scores of different age groups were normally distributed and compared using ANOVA, with a *p* value of 0.05.

## Results

No significant differences between the subscales for restricted repetitive and stereotyped patterns of behaviour (attention to detail and attention switching) and the other subscales representing communication and social interaction were found. On the subscale of imagination there was a significant difference between the different age groups, especially between the group of 18–30 years and > 60 years. This, however, was not the focus of this investigation.

**Fig. 1:**
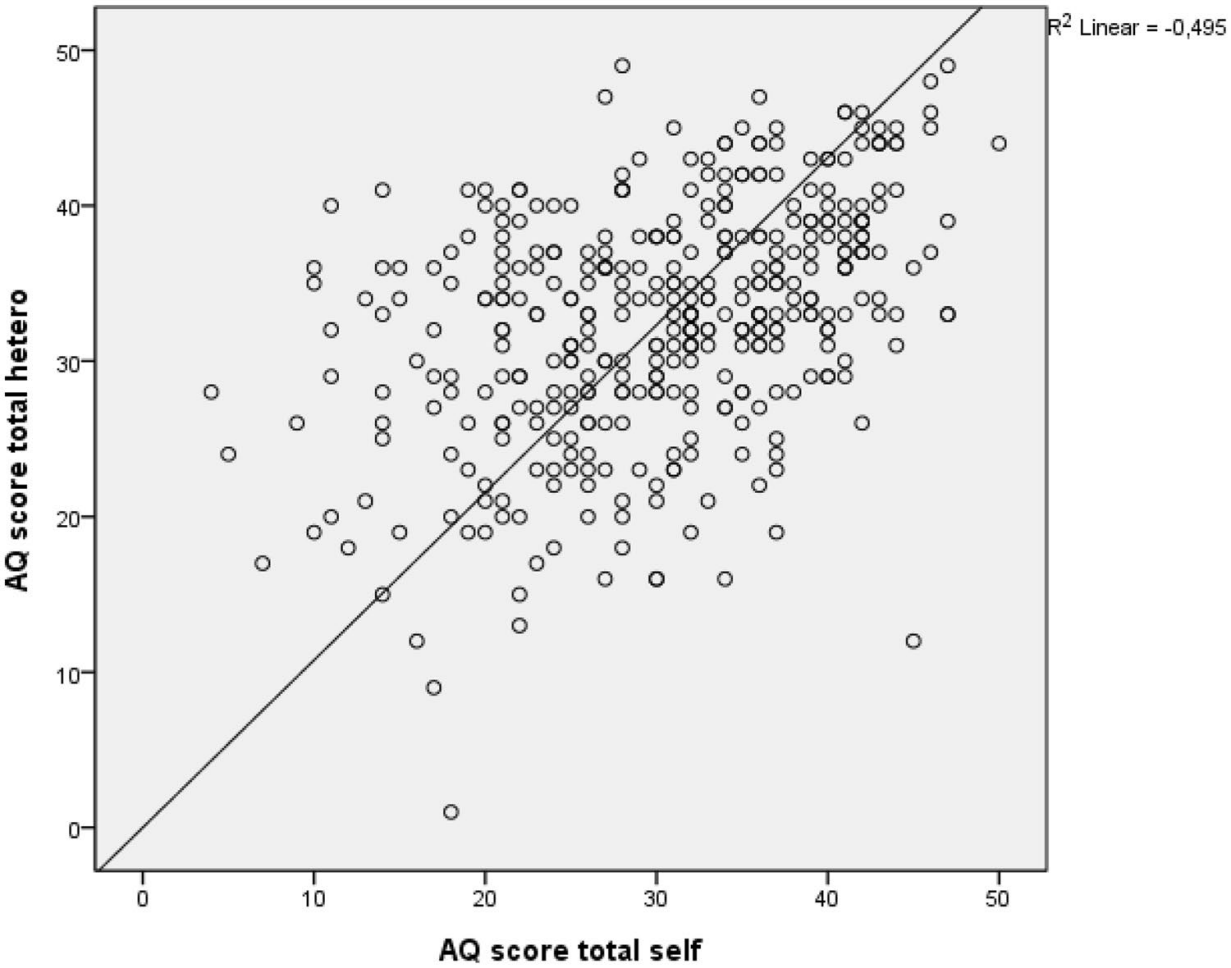
Correlation of AQ score self-report and AQ score reported by spouses

Significant differences were found between the subscales and total AQ between the self-reported AQ’s and the AQ reported by spouses, except for the imagination subscales and the communication and social skills subscales reported by spouses (Table 1). For example: the total AQ scores of the self-reported group were 30.59 (SD 8.80) these of the group reported by spouses were 32.69 (SD 7.73) (Table 1). The differences, however, are too little to be of clinical relevance, the AQ scores based on self-report corresponded actually remarkably well with those from their spouses. Significant correlations were found between the self-reported AQ scores and these reported by spouses (Fig. 1).

## Discussion

This study shows two major findings. First, the main traits of an ASD in patients referred to an outpatient University clinic, as represented by the subscales of the AQ, are stable between the different adult age groups. The big strength of this study is that this was found in a large cohort of patients. This indicates that the distribution of symptoms is stable during adult life. However this does not mean that the problems experienced by these symptoms are equal per age group. Clinically this finding indicates that autism experts should take into account that basic principles of interventions which are effective in younger age groups can also be applied in later age groups. However, our study is based on referrals as is custom in the Health Care system in The Netherlands. Variations between countries are considerable, and our advises should be tailored to the organization of Health Care per country.

This all implies that the hypothesis that differences between groups at different ages, would be found for social and communication domains, but not for the repetitive and restrictive behaviours, could not be supported.

Second, high correlations were found between the AQ scores of patients and their spouses, even though the main AQ scores marginally, but significantly differ between them. As a group, patients and spouses see a remarkably equal pattern of symptoms. Spouses seem to be capable to do reliable observations of symptoms of their relatives. Both spouses and patients do score above the cut-off score of 26 as proposed in the clinical study of Woodbury-Smith et al. (2005). This could indicate that the AQ can also be used as a questionnaire reported by spouses, which already has been validated in an adolescent and child’s group, but, to our knowledge, has never been validated for the adult group. In this way the AQ, reported by spouses, can be of extra value in the screening procedure for an ASD. However, first a validation study is necessary.

The most important limitation of the study is that it is not longitudinal. Nothing can be concluded about the differences in symptom distribution during the life-span of patients with ASD.

Furthermore the included patients were referred to a university hospital; clinically this can be a selection of patients. Also it is not representative for the general population. Not all questionnaires were completed by both patients and spouses. Sometimes no spouses were available, or questionnaires were not submitted for the data-collection. Nevertheless there were a substantial number of 380 patients were both questionnaires were filled in. The cohorts of patients older than 70 years are relatively small in comparison to the other groups.

A further limitation is that the AQ has not the validity that it can be used as a diagnostic tool, it is validated as a screenings instrument. It is not clear if the subscales can be used to compare the different symptom clusters between different age groups. Since there is very little information on the differences of symptoms between different ages, this study tries to add more information on the subject using these subscales on the AQ.

It is unclear what can be concluded form the significant difference between the imagination subscale between the younger and older age groups, in which older age groups have higher scores on the imagination subscales than younger age groups. It could be possible that this is a normal variance between age groups, although this had never been published.

It can be discussed whether attention to detail and attention switching relate to stereotypic and repetitive behaviors. Previous studies have shown that inhibitory control and attentional flexibility predicts stereotyped behavior in everyday life (Mostert-Kerckhoffs et al. 2015).

Overall, the conclusions of this study are that the symptoms of the group of patients included are stable between the different adult age groups. Furthermore the AQ scores of patients and spouses are remarkably comparable. It is interesting to investigate the distribution of symptoms during life using the AQ, but therefore a longitudinal study is necessary. In such a study the level of functioning of the participants should also be included.

## References

[CR1] Auyeung B, Baron-Cohen S, Wheelwright S, Allison C (2008). The Autism Spectrum Quotient: Children’s Version (AQ-Child). Journal of Autism and Developmental Disorders.

[CR2] Baron-Cohen S, Hoekstra RA, Knickmeyer R, Wheelwright S (2006). The Autism-Spectrum Quotient (AQ): Adolescent version. Journal of Autism and Developmental Disorders.

[CR3] Baron-Cohen S, Wheelwright S, Skinner R, Martin J, Clubley E (2001). The Autism-Spectrum Quotient (AQ): Evidence from Asperger syndrome/high-functioning autism, males and females, scientists and mathematicians. Journal of Autism and Developmental Disorders.

[CR4] Billstedt E, Gillberg IC, Gillberg C (2007). Autism in adults: Symptom patterns and early childhood predictors: Use of the DISCO in a community sample followed from childhood. Journal of Child Psychology and Psychiatry.

[CR5] Broadbent J, Galic I, Stokes MA (2013). Validation of autism spectrum quotient adult version in an Australian sample. Autism Research and Treatment.

[CR6] Chowdhury M, Benson BA, Hillier A (2010). Changes in restricted repetitive behaviors with age: A study of high-functioning adults with Autism Spectrum Disorders. Research in Autism Spectrum Disorders.

[CR7] Gillespie-Lynch K, Sepeta L, Wang Y, Marshall S, Gomez L, Sigman M (2012). Early childhood predictors of the social competence of adults with autism. Journal of Autism and Developmental Disorders.

[CR8] Hoekstra RA, Bartels M, Cath DC, Boomsma DI (2008). Factor structure, reliability and criterion validity of the Autism-Spectrum Quotient (AQ): A study in Dutch population and patient groups. Journal of Autism and Developmental Disorders.

[CR9] Howlin P, Savage S, Moss P, Tempier A, Rutter M (2014). Cognitive and language skills in adults with autism: A 40-year follow-up. Journal of Child Psychology and Psychiatry.

[CR10] Lepage J-F, Lortie M, Taschereau-Dumouchel V, Théoret H (2009). Validation of French-Canadian Versions of the Empathy Quotient and Autism Spectrum Quotient. Canadian Journal of Behavioural Science.

[CR11] Levy A, Perry A (2011). Outcomes in adolescents and adults with autism: A review of the literature. Research in Autism Spectrum Disorders.

[CR12] Magiati I, Tay XW, Howlin P (2014). Cognitive, language, social and behavioural outcomes in adults with autism spectrum disorders: A systematic review of longitudinal follow-up studies in adulthood. Clinical Psychology Review.

[CR13] McGovern CW, Sigman M (2005). Continuity and change from early childhood to adolescence in autism. Journal of Child Psychology and Psychiatry.

[CR14] Mostert-Kerckhoffs MA, Staal WG, Houben RH, de Jonge MV (2015). Stop and change: Inhibition and flexibility skills are related to repetitive behavior in children and young adults with autism spectrum disorders. Journal of Autism and Developmental Disorders.

[CR24] Newschaffer CJ, Croen LA, Daniels J, Giarelli E, Grether JK, Levy SE (2007). The epidemiology of autism spectrum disorders. Annual Review of Public Health.

[CR15] Pisula E, Kawa R, Szostakiewicz L, Lucka I, Kawa M, Rynkiewicz A (2013). Autistic traits in male and female students and individuals with high functioning autism spectrum disorders measured by the Polish version of the Autism-Spectrum Quotient. PLoS ONE.

[CR16] Seltzer MM, Krauss MW, Shattuck PT, Orsmond G, Swe A, Lord C (2003). The symptoms of autism spectrum disorders in adolescence and adulthood. Journal of Autism and Developmental Disorders.

[CR17] Seltzer MM, Shattuck P, Abbeduto L, Greenberg JS (2004). Trajectory of development in adolescents and adults with autism. Mental Retardation and Developmental Disabilities Research Reviews.

[CR18] Sizoo BB, Horwitz EH, Teunisse JP, Kan CC, Vissers C, Forceville E (2015). Predictive validity of self-report questionnaires in the assessment of autism spectrum disorders in adults. Autism.

[CR19] Sonie S, Kassai B, Pirat E, Bain P, Robinson J, Gomot M (2013). The French version of the autism-spectrum quotient in adolescents: A cross-cultural validation study. Journal of Autism and Developmental Disorders.

[CR20] Taylor JL, Seltzer MM (2010). Changes in the autism behavioral phenotype during the transition to adulthood. Journal of Autism and Developmental Disorders.

[CR21] Taylor MJ, Gillberg C, Lichtenstein P, Lundstrom S (2017). Etiological influences on the stability of autistic traits from childhood to early adulthood: Evidence from a twin study. Molecular Autism.

[CR22] Wakabayashi A, Baron-Cohen S, Wheelwright S, Tojo Y (2006). The Autism-Spectrum Quotient (AQ) in Japan: A cross-cultural comparison. Journal of Autism and Developmental Disorders.

[CR23] Woodbury-Smith MR, Robinson J, Wheelwright S, Baron-Cohen S (2005). Screening adults for Asperger Syndrome using the AQ: A preliminary study of its diagnostic validity in clinical practice. Journal of Autism and Developmental Disorders.

